# Ultrasound Evaluation of Sarcopenia in Patients with Hepatocellular Carcinoma: A Faster and Easier Way to Detect Patients at Risk

**DOI:** 10.3390/diagnostics14040371

**Published:** 2024-02-08

**Authors:** Giorgio Esposto, Raffaele Borriello, Linda Galasso, Fabrizio Termite, Irene Mignini, Lucia Cerrito, Maria Elena Ainora, Antonio Gasbarrini, Maria Assunta Zocco

**Affiliations:** CEMAD Digestive Disease Center, Fondazione Policlinico Universitario “A.Gemelli” IRCCS, Università Cattolica del Sacro Cuore, 20123 Rome, Italy; giorgio.esposto@guest.policlinicogemelli.it (G.E.); raffaele.borriello@guest.policlinicogemelli.it (R.B.); linda.galasso0817@gmail.com (L.G.); fabrizio.termite@libero.it (F.T.); irene.mignini@guest.policlinicogemelli.it (I.M.); lucia.cerrito@policlinocogemelli.it (L.C.); mariaelena.ainora@policlinicogemelli.it (M.E.A.); antonio.gasbarrini@unicatt.it (A.G.)

**Keywords:** hepatocellular carcinoma, ultrasound, sarcopenia

## Abstract

The condition of sarcopenia, defined as a progressive loss of musculoskeletal mass and muscular strength, is very common in patients with hepatocellular carcinoma (HCC) and presents a remarkable association with its prognosis. Thus, the early identification of sarcopenic patients represents one of the potential new approaches in the global assessment of HCC, and there is increasing interest regarding the potential therapeutic implications of this condition. The gold standard for the quantification of muscle mass is magnetic resonance imaging (MRI) or computed tomography (CT), but these techniques are not always feasible because of the high-cost equipment needed. A new possibility in sarcopenia identification could be muscle ultrasound examination. The measurement of specific parameters such as the muscle thickness, muscular fascicles length or pennation angle has shown a good correlation with CT or MRI values and a good diagnostic accuracy in the detection of sarcopenia. Recently, these results were also confirmed specifically in patients with chronic liver disease. This review summarizes the role of imaging for the diagnosis of sarcopenia in patients with HCC, focusing on the advantages and disadvantages of the diagnostic techniques currently validated for this aim and the future perspectives for the identification of this condition.

## 1. Introduction

Hepatocellular carcinoma (HCC) represents the most common primary liver cancer and a current global health challenge, being the fifth most prevalent solid tumor worldwide and the second cause of tumor-related death in men [1]. Its incidence is constantly growing, and it is expected that by 2025, more than one million individuals will be affected by liver cancer annually [2]. Despite the major therapeutic advances of the last few decades, HCC still presents a generally poor prognosis, with a global 5-year survival rate of about 16% [3,4]. A crucial issue in the actual scenario is represented by the identification of prognostic biomarkers able to provide an early identification of patients at a high risk of death or therapeutic failure. In this context, the evaluation of nutritional status and, in particular, of muscular mass impairment (sarcopenia), has recently emerged as a possible new instrument in the management of this disease [5].

Sarcopenia can be defined as a progressive and generalized disorder which results in a reduced muscle quantity and muscle strength [6], increasing the likelihood of adverse outcomes such as reduced mobility, falls, fractures and disability [6,7]. It is possible to distinguish “primary” or age-related sarcopenia, when no apparent causes or underlying factors are evident [6], and “secondary” sarcopenia, when this condition arises in the context of a systemic, inflammatory or malignant disease [6]. Secondary sarcopenia is very common in patients with HCC. According to a recent meta-analysis which included 48 studies and 8959 patients with HCC, about 42% of the whole cohort was sarcopenic [8]. Moreover, it is known that, in patients with HCC, the presence of sarcopenia predicts negative outcomes in each stage of the disease [9], and is associated with adverse events and poor prognosis in patients undergoing liver resection [10,11], liver transplant [12,13], trans-arterial chemoembolization (TACE) [14,15,16,17], trans-arterial radioembolization (TARE) [18], percutaneous radio-frequency ablation (RFA) [19,20,21,22] and systemic therapy [23,24]. Thus, the early diagnosis of this condition could be useful for the identification of patients with a worse prognosis and could represent a new therapeutic target to improve the overall survival and the quality of life of these patients [5].

While the assessment of muscle strength and physical performance can be performed through several cheap and easily available clinical tests such as the handgrip strength test, the chair stand test and the gait speed velocity measurement [6,7], the evaluation of the muscle mass quantity requires the calculation of individual parameters which can be identified through specific imaging techniques. To date, the gold standard for the radiological diagnosis of sarcopenia is the quantification of muscle mass through a computed tomography scan (CT) or magnetic resonance imaging (MRI) [6,25]. Furthermore, the evaluation of the total skeletal muscle index (SMI) through the quantification of the muscular area at the level of the L3 vertebral body has shown a good capability to predict the total muscle mass and body composition [6,25]. This parameter has been extensively used for the assessment of sarcopenia in patients with HCC [8,26]. However, the routinary use of CT and MRI for the detection of sarcopenia is limited by the high costs of these techniques, the use of ionizing radiation for CT scans and the need of specific equipment and trained specialists for both methods [6,27]. Moreover, CT and MRI need to be scheduled in advance and are often associated with a long waiting list. An alternative, easily available and safe technique to assess muscle mass is dual-energy X-ray absorptiometry (DXA), which consists of a whole-body scan through a specific machine which uses a very low dose of ionizing radiation [27]. DXA is considered a simple, valuable, cheap and reproducible method to assess muscle mass with a strong correlation with the results of CT and MRI [25,27]. However, this technique is affected by individual factors such as body thickness, hydration status and water retention, making it unreliable in pathologic conditions associated with fluid retention such as ascites [27], and, as a consequence, in some patients with HCC. Bioelectrical impedance analysis (BIA) represents another feasible technique able to estimate the muscle mass quantity with the application of a low-intensity electrical current through the whole body [28]. Even if it has the advantages of being cheap, accurate, easy to perform and portable, this technique is also affected by individuals’ hydration status, and seems to be associated with a significant grade of variability among different health conditions, specific populations and different equipment [6,27,28].

Among the emerging methods for the evaluation of muscle mass and the diagnosis of sarcopenia is muscle ultrasound (MUS). It is a cheap, non-invasive and easily available technique already validated for the diagnosis of several musculoskeletal conditions [29], representing a reliable instrument for the evaluation of muscle quantity and quality [6,27,30]. Compared to CT and MRI, MUS can be performed at the bedside, requires a significantly shorter time and does not require the presence of a radiology technician. Moreover, MUS, unlike CT, does not expose the patient to potentially harmful radiations. MUS allows for the measurements of several variables, such as the muscle thickness (MT), cross-sectional area (CSA), fascicle length (FL), pennation angle (PA) and echogenicity index (EI) [30,31], the alterations of which have shown a strong correlation with muscular performance [32,33] and structure [27,30], with results similar to those obtained by the gold standards CT or MRI [31]. Even if the majority of data concerning the performance of MUS have been collected in the geriatric population [34], its usefulness has been recently studied also in patients affected by chronic liver disease [35] and hepatocellular carcinoma [36], with promising results in terms of the reliability and prognostic value.

To the best of our knowledge, this is the first review that explores the state of the art regarding the application of MUS for the diagnosis of sarcopenia in patients with HCC, evidencing the potential benefits of its routinary use in clinical practice as well as the pitfalls and limits of its application.

## 2. Pathophysiology of Sarcopenia in HCC Patients

The biological basis of sarcopenia in HCC patients is complex and multifactorial and depends both on the cancer itself and upon factors that are not tumor related. Cancer induces systemic inflammation by cytokine production (IL-1, IL-6, TNF-a, IFN-y) and due to reactive oxygen species (ROS) imbalance; this inflammatory microenvironment favors proteolysis and increases gluconeogenesis and insulin resistance. Insulin resistance facilitates fat deposition between muscle fibers, resulting in myosteatosis [37].

**Figure 1:**
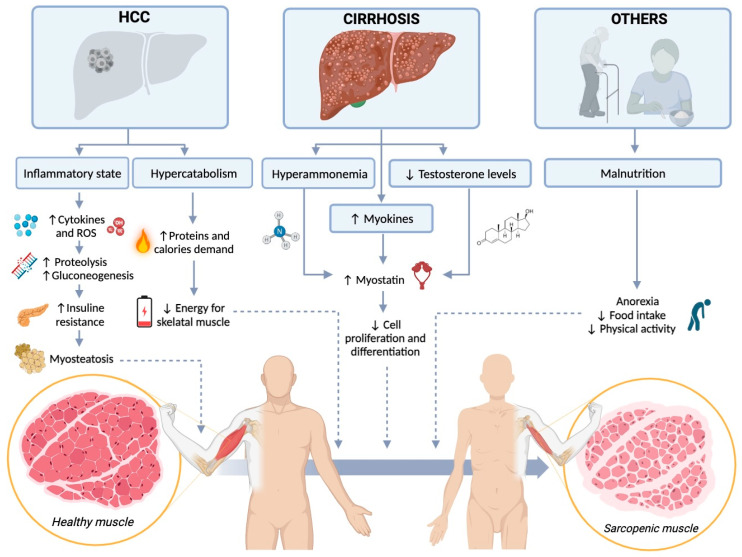
Biological basis of sarcopenia in hepatocellular carcinoma. Created with BioRender.com. HCC, hepatocellular carcinoma; ROS, reactive oxygen species.

Patients with cirrhosis-related HCC display the condition of cellular starvation and a hypermetabolic state that raises the demand of proteins and calories. The increased usage of amino acids results in a reduced supply of energy sources for skeletal muscles and therefore induces muscle degradation. In this context, malabsorption and inadequate nutrient intake potentially contribute to sarcopenia development. The nutritional deficiencies reduce the muscle anabolic activity and therefore can further worsen muscle wasting. Moreover, in cirrhotic patients, hyperammonemia and low testosterone levels are also involved with muscle wasting. This hormonal imbalance is responsible for the upregulation of myokines (myostatin, IL-6 and follistatin). Follistatin, a myostatin, is an inhibitor of the TGF-β superfamily related to the tumor size and stage, and it plays an oncogenic role in hepatocarcinogenesis. This complex interplay between muscle wasting and carcinogenesis could be a future therapeutic target for sarcopenia [38] (Figure 1).

## 3. Ultrasound Assessment of Sarcopenia

**Figure 2:**
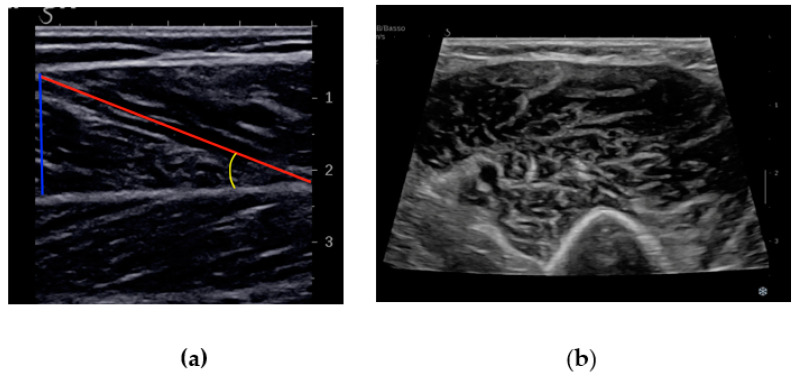
(**a**) A longitudinal US scan of the gastrocnemius muscle showing muscle thickness (blue line), fascicle length (red line) and pennation angle (yellow line); (**b**) a transverse US scan of biceps muscle showing cross-sectional area.

MUS provides an easy-to-use, noninvasive and point-of-care tool for assessing sarcopenia with the advantage of multiple sequential assessments. It allows one to estimate the muscle mass and to evaluate the distribution of adipose and fibrous tissue within skeletal muscle. The European Geriatric Medicine Society (EuGMS) sarcopenia group recently proposed a consensus protocol on the ultrasound evaluation of muscle mass, which involves the measurement of MT, CSA, FL, PA and EI [39] (Figure 2). In particular, MUS can provide a detailed evaluation of the quantitative and qualitative parameters of specific anatomical sites.

### 3.1. Quantitative Ultrasound Parameters

The thickness of specific muscle groups provides valuable information on the muscle size and composition since it directly reflects the degree of muscle atrophy. A reduction in the MT indicates a loss of muscle mass. This parameter is often measured in the quadriceps, where it has been shown to be a good predictor of the muscle volume, but it can be targeted to other muscle groups as well.

Another interesting parameter is the CSA, defined as the area of a muscle in a transverse plane. Both the CSA and MT provide numerical data, allowing for accurate comparisons over time and between individuals. It has been shown that individuals with a significant reduction in the MT and CSA may be at a higher risk for complications related to sarcopenia [40]. Moreover, in clinical settings, both parameters could serve as accurate endpoints for evaluating treatment efficacy [40].

With MUS, it is also possible to evaluate the PA and FL, defined as the orientation of muscle fibers with respect to the tendon and the length of muscle fibers within a muscle, respectively. The PA has a direct impact on muscle strength: muscles with a higher PA can generate greater force due to the increased number of fibers that can be packed into a given cross-sectional area. Hence, muscles with a parallel fiber arrangement, like the biceps brachii, usually have an angle close to zero since they are designed for speed and range of motion. On the other hand, muscles with an oblique fiber arrangement designed for force generation, like the gastrocnemius, have a larger PA that can range from 10 to 30 degrees or more. Alterations in the PA may be indicative of structural changes associated with sarcopenia, offering additional insights into muscle health. Ultrasound can easily measure this angle, providing information about muscle architecture [34]. The measurement of the FL helps to define the architectural arrangement of muscle tissues This parameter has a direct impact on muscle strength: the longer the fascicle, the greater the mechanical advantage and hence the force generated. Therefore, a reduced FL may lead to diminished muscle strength and functional impairment. This evaluation is particularly relevant in predicting the impact of sarcopenia on activities of daily living and the overall quality of life [34,41].

### 3.2. Qualitative Ultrasound Parameters

**Figure 3:**
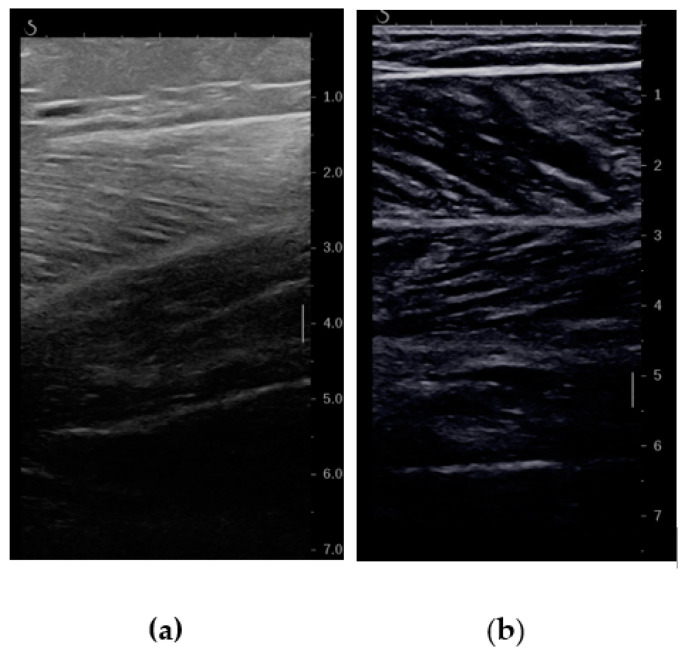
Differences in echogenicity of gastrocnemius. (**a**) High echogenicity index (EI) in an old patient with myosteatosis and sarcopenia. (**b**) Normal EU in a young patient.

While assessing sarcopenia, emphasis has traditionally been placed on evaluating muscle mass for diagnosis. However, it is evident that reduced muscle mass alone does not comprehensively account for the decline in muscle strength. Additionally, age-related changes in muscle strength outpace those related to muscle mass, suggesting the potential significance of qualitative muscular changes alongside quantitative parameters. Within the realm of ultrasound characteristics, the Echo Intensity (EI) emerges as a critical indicator reflecting variations in muscle quality. The EI, denoting the brightness or darkness observed in ultrasound images of muscle tissue, offers valuable insights into the distribution of adipose and fibrous tissues within skeletal muscles. Notably, elevated levels of intramuscular fat correspond to increased muscle brightness, termed hyperechogenicity, in ultrasound imaging (see Figure 3). Studies have established a negative correlation between the EI of the quadriceps femoris and quadriceps strength [42].

### 3.3. Anatomical Sites for Muscle Ultrasound

MUS evaluation is usually performed with the patient in a supine position, applying a gentle pressure with a high-frequency (5–14 MHz) linear array probe. The quadriceps and hamstring muscles are commonly assessed due to their accessibility and their central role in mobility and daily activities. In patients with limited lower limb mobility, the biceps brachii, triceps brachii and transversus abdominis are frequently evaluated. Among the previously described MUS parameters, the MT of the gastrocnemius, rectus femoris, tibialis anterior and soleus have shown a moderate accuracy for the diagnosis of sarcopenia [40]. Similar results were obtained for the CSA of the rectus femoris and biceps brachii and the FL of the gastrocnemius [40,43]. A complete ultrasound evaluation should be targeted to all the above-mentioned muscle groups.

### 3.4. Advantages of Ultrasound in the Diagnosis of Sarcopenia

The non-invasive nature of MUS makes it a safe and easily accessible tool for the regular monitoring and follow-up assessment of sarcopenia. First of all, patients can undergo real-time evaluation without exposure to ionizing radiation or the need for contrast agents, unlike CT and MRI. This is important, especially in patients with renal impairment. Moreover, MUS is a rapid and cost-effective method that can be performed at the patient’s bedside with widely available equipment, unlike standard imaging techniques that require a longer time of evaluation, sometimes with patients in uncomfortable positions, and more expensive instruments [40,43]. 

Finally, unlike traditional static imaging, real-time ultrasound allows for the direct observation of muscle at rest and in motion, giving immediate feedback on the muscle quality [44] and performance [45]. The possibility to perform a dynamic evaluation is important not only in the differential diagnosis with other potential causes of muscle weakness but also to evaluate the impact of sarcopenia on a patient’s daily life [46,47]. All the above-mentioned advantages make MUS a useful option for assessing and monitoring sarcopenia over time in healthcare settings. A comparison between MUS and other techniques for the diagnosis of sarcopenia can be found in Table 1.

### 3.5. New Technologies: Shear Wave Elastography and Quantitative Ultrasound

**Figure 4:**
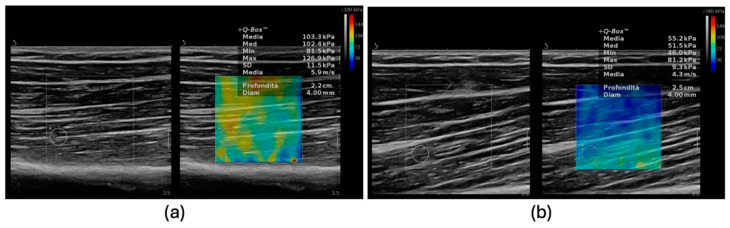
Differences in muscle stiffness evaluated by SWE. (**a**) Elevated stiffness in an old patient with myosteatosis and sarcopenia (103.3 kPa). (**b**) Lower stiffness in a young patient (55.2 kPa). SWE, shear wave elastography, kPa, kilopascal.

Recently, novel US based techniques have been evaluated as adjunctive tools in the diagnosis of sarcopenia. Preliminary evidence suggests a possible role of shear wave elastography (SWE), a method used to assess tissue stiffness, in the assessment of muscle quantity and quality [48] (Figure 4). SWE velocities have shown a significantly lower value in sarcopenic patients and have been positively correlated with grip strength [48]. These results could be related to the muscle structural rearrangements that occur in sarcopenic patients with increased adipose tissue deposition and fibrosis.

Another emergent technology is represented by quantitative ultrasound that aims to estimate the quantitative characteristics of analyzed tissues through the evaluation and measurement of backscattered signals [49]. Indeed, the analysis and quantification of acoustic scattering parameters associated with the acoustic impedance, for example, the backscatter coefficient or scatterer size, is able to provide an estimation of the microstructural and elastic properties of tissues [49,50]. This technology is far from clinical application in muscle ultrasound because the aforementioned quantitative variables cannot be easily applied to anisotropic tissues, like skeletal muscle. A recently introduced geometric model, published by Santoso et al., could overcome these limits [51].

## 4. Sarcopenia in HCC Patients

As described in the Introduction, the early detection and monitoring of sarcopenia in patients with HCC can significantly influence clinical decision-making. In particular, the identification of individuals at a higher risk for sarcopenia-related complications could drive targeted interventions, including nutritional support and physical therapy. Furthermore, clinicians can adjust treatment regimens, including chemotherapy dosages, to prevent potential muscle-related side effects. This personalized approach enhances overall patient care and improves treatment outcomes [52].

The role of MUS has been extensively studied in patients with liver cirrhosis. In 2019, Hari et al. demonstrated that the psoas muscle diameter was significantly related to hospitalization and mortality in patients with decompensated liver cirrhosis [35]. More recently, Dhariwal et al. evaluated patients with liver cirrhosis and sarcopenic obesity and demonstrated a positive correlation of different MUS parameters with CT scan-derived SMI [53].

While there is large consensus on the utility of MUS for the assessment of sarcopenia in patients with liver disease, to date, there is little evidence concerning the role of MUS in patients with HCC [36]. In this specific clinical setting, the available data refer to standard imaging techniques and are focused mainly on preoperative risk assessment or the prediction of prognosis in patients undergoing treatment. It is known that sarcopenia, characterized by the loss of muscle mass and function, is associated with increased surgical complications, longer hospital stays, and poorer postoperative outcomes [54,55]. The possibility to assess muscle mass could be useful to evaluate morphological and functional changes over time. This is especially relevant for patients undergoing major surgeries with potential impacts on muscle health.

In 2015, Kobayashi et al. retrospectively analyzed the CT scans of 241 patients undergoing primary hepatectomy for HCC. They evaluated post-operative changes in the intramuscular adipose tissue content (IMAC) and the psoas muscle mass index (PMI) and their correlation with HCC recurrence during follow-up. In a multivariate analysis, the increase in the IMAC 6 months after hepatectomy was significantly correlated with HCC recurrence (OR = 3.713; *p* = 0.024) in patients with a normal preoperative IMAC [56].

The role of the preoperative assessment of sarcopenia was evaluated by Hamaguchi et al. in a retrospective study performed in 492 patients undergoing hepatectomy for HCC. They analyzed the impact of preoperative IMAC assessed by CT scan on the development of surgical complications after hepatectomy. An increased IMAC emerged as an independent risk factor for major postoperative complications, showcasing an odds ratio (OR) of 1.580 (*p* = 0.049). In particular, the authors found a higher susceptibility to infections, with an OR of 1.903 (*p* = 0.021) [57].

In a recent study focusing on the impact of sarcopenia on the postoperative outcomes of HCC patients [10], a total of 155 patients underwent evaluation through handgrip strength and chair stand tests, physical performance, and CT scans. The patients were categorized into three groups based on muscle mass and strength. The baseline data and postoperative changes were compared. The primary outcome was the occurrence of major postoperative complications, while the secondary outcome was the 90-day re-admission rate. The group exhibiting a diminished muscle mass and strength post-surgery experienced a higher incidence of major complications, increased blood transfusion rates, elevated hospitalization costs (*p* = 0.001),and prolonged hospital stays (*p* < 0.001). However, no significant differences in the 90-day re-admission rates were observed among the three groups. Notably, sarcopenia (hazard ratio: 10.735; 95% CI: 2.547–45.244; *p* = 0.001) and open surgery (hazard ratio: 4.528; 95% CI: 1.425–14.387; *p* = 0.010) emerged as independent risk factors for the occurrence of major complications [10].

The overall influence of sarcopenia on the post-operative prognosis of HCC was recently summarized by Kong and colleagues [58]. They analyzed 30 studies, with a total of 7352 HCC patients (2695 in the sarcopenia group and 4657 in the non-sarcopenia group) who had undergone curative surgical resection. The meta-analysis performed on 28 out of the 30 studies indicated that the patients with sarcopenia had a significantly lower overall survival (HR = 2.20; 95% CI, 1.88–2.58; *p* < 0.01) [58]. These findings confirm the considerable importance of identifying and correcting sarcopenia in HCC patients who are candidates for surgery.

The role of sarcopenia developed after surgery was explored by Yang et al. in 62 patients treated with curative hepatectomy and adjuvant TACE. The loss of skeletal muscle (in terms of a low SMI measured by CT) during the 6 months following treatment was predictive of worse liver-related survival [59].

The impact of sarcopenia in patients with HCC is not only limited to the prognosis of subjects undergoing surgery, but it also has possible implications in the outcomes of other treatments, including pharmacological therapy.

Wu et al. [60] investigated if sarcopenia defined with a CT scan and evaluated in different muscles can predict the prognosis of HCC after radioembolization. The pre-treatment assessment included the examination of the abdominal muscle, psoas muscle and paraspinal muscle. Each muscle was analyzed to determine its potential as a prognostic factor for OS. Interestingly, sarcopenic patients identified by the psoas muscle had a notably inferior OS compared to those without sarcopenia, while sarcopenia defined by the abdominal and paraspinal muscles was not associated with significant differences in prognosis. Upon adjusting for clinical variables, the presence of sarcopenia defined by the psoas muscle remained an independent predictor for a poor OS (HR: 1.899, 95% CI: 1.087–3.315) [60].

Nam et al. [61] explored the effects of sarcopenia in HCC patients treated with TARE. In this multi-center retrospective study, sarcopenia was assessed by CT. A multivariate Cox regression analysis revealed that a low skeletal muscle mass (LSMM) was independently associated with a poor OS (HR, 1.36; 95% CI, 1.00–1.85, *p* = 0.05). These findings suggest that pre-treatment LSMM could serve as a surrogate biomarker for identifying TARE candidates [61].

The impact of sarcopenia in patients selected for TACE was studied by Loosen and colleagues [15]. As in the previously cited studies, the authors defined sarcopenia by a CT scan; the parameters evaluated were the SMI, median muscular attenuation (MMA), bone mineral density (BMD) and the visceral and subcutaneous fat area. The results were correlated with the tumor response to TACE and the patients’ outcome. The pre-interventional SMI turned out to be an independent prognostic factor for the clinical outcome (HR: 0.899, 95% CI 0.827–0.979, *p* = 0.014). These findings are consistent with those of Chien et al. [62], who evaluated sarcopenia by CT before the first TACE session. Once again, a multivariate analysis demonstrated that sarcopenia was an independent poor prognostic factor for the overall survival in HCC patients receiving TACE.

In a retrospective study by Kobayashi et al. conducted on 102 patients treated with trans-arterial treatments, the authors evaluated the SMI through a CT scan both at the baseline and 6 months after the treatment [63]. Notably, they found that the OS did not differ significantly among the patients with or without sarcopenia at baseline but was significantly lower in those who showed a higher muscle loss at 6 months.

In patients undergoing systemic therapy with Sorafenib, a lower muscle mass at baseline and rapid muscle wasting during treatment were associated with a poorer prognosis in terms of the OS and early therapeutic discontinuation [64,65,66,67,68]. In a retrospective study conducted by Cheng et al. [69] on 385 patients with disease progression after Sorafenib, a poorer prognosis was observed in those with a lower muscle mass at the time of therapeutic failure. Notably, in this group of patients, the gain of muscle mass after therapeutic failure was associated with a higher post-progression survival. The effective benefit in the reversal of this condition suggests the potential role of a nutritional therapeutic approach [69].

Xiong and colleagues [70] recently conducted a study exploring the prognostic relevance of sarcopenia in patients undergoing immune checkpoint inhibitor therapy. Utilizing CT scans, sarcopenia was assessed by computing various parameters such as the SMI, visceral adipose tissue index, subcutaneous adipose tissue index (SATI) and total adipose tissue index. A multivariate analysis demonstrated that both the SATI (HR 0.251; 95% CI 0.109–0.577; *p* = 0.001) and the presence of sarcopenia (sarcopenia vs. no sarcopenia; HR 2.171; 95% CI 1.100–4.284; *p* = 0.026) independently served as prognostic indicators for the OS [70].

Imai et al. [71] studied the effects of lenvatinib or sorafenib treatment on body composition and how these changes could affect OS. They evaluated the SMI, subcutaneous and visceral adipose tissue indices before treatment, after three months and at treatment discontinuation or the last observation. Both the pre-treatment SMI and its decrease during treatment were independent prognostic factors for HCC [71].

In patients with HCC treated with lenvatinib, Uojima et al. observed an association between sarcopenia and a worse prognosis not only in terms of the OS and treatment failure, but also in terms of the treatment tolerability and severe adverse events [72].

Another recent study conducted by Luo et al. investigated the prognostic value of a low PMI in patients with HCC undergoing combination therapy with immune checkpoint inhibitors and tyrosine kinase inhibitors. Sarcopenia was an independent negative prognostic factor in the long-term outcomes, being associated with the risk of death at both 1 year and at the end of the follow-up [73].

The investigation by Oura et al. [24] delved into the correlation between sarcopenia and the prognosis of patients with hepatocellular carcinoma (HCC) treated using Atezolizumab plus Bevacizumab. Sarcopenia was assessed through the skeletal muscle index (SMI) defined by a bioelectrical impedance analysis (BIA) and grip strength measurements. The median progression-free survival was observed to be 4.7 months (range: 0.4–26.4) in the sarcopenia group and 10.6 months (range: 1.1–24.5) in the non-sarcopenia group. Upon conducting a multivariate analysis, sarcopenia exhibited a significant association with the OS, notably concerning the occurrence of adverse effects and decreased liver function.

These results were analogous to those of a recent multicenter retrospective study conducted by Hiraoka et al. [74].

Finally, a study conducted by Yang et al. investigated the correlation among sarcopenia and clinical outcomes in patients undergoing stereotactic body radiotherapy [75]. In this study, the loss of muscle mass after radiotherapy, rather than the presence of pre-therapy sarcopenia, was predictive of poor survival and liver toxicity.

To our knowledge, there have been no studies performed with MUS to assess the impact of sarcopenia for risk stratification in patients with HCC undergoing surgery or other treatments. On the other hand, only one recent study evaluated the role of MUS in the diagnosis of sarcopenia in patients with HCC [36]. In this study, the ultrasound evaluation of the MT and subcutaneous fat of the lower limbs was compared to the SMI measured by CT in 30 patients with HCC. The assessment encompassed six distinct muscles in the lower limbs: the right gastrocnemius medial head, right gastrocnemius lateral head, right soleus, left gastrocnemius medial head, left gastrocnemius lateral head and left soleus.

The authors found no significant correlation between the SMI and MT, but only between the SMI and left subcutaneous fat thickness (r = 0.406, *p* = 0.026). It is unclear why these results differ from the results of patients not affected by HCC, in whom there is a large consensus on the utility of ultrasound in sarcopenia assessment (Table 2). The authors suggest that in HCC patients, the progression of sarcopenia is worse in the lower limbs than the trunk. This could imply that lower limb muscle atrophy could not correlate with the SMI in those patients with a normal SMI.

The cited articles do not represent the entirety of studies on HCC and sarcopenia. They were specifically chosen to highlight the impact of sarcopenia in HCC patients and the need for new techniques to simplify its evaluation. MUS could aid in this field of the study, but needs more evidence before it can be routinely applied into clinical practice.

## 5. Discussion

Sarcopenia is a major actor in the natural history of HCC, as highlighted by Liu et al. in a recent meta-analysis on 8959 HCC patients in which 42% of the whole cohort was sarcopenic [8]. As clearly discussed above, sarcopenia has a negative impact in each stage of the disease and implies worse prognosis in terms of the therapeutic response, OS [9,15,24,60,61,62,70,71], surgical complications [10,56,57] and HCC recurrence [56].

Furthermore, the wasting of muscle mass after treatments has been shown to affect equally the global prognosis and survival [63,75]. In this context, the reversal of this condition could potentially benefit the OS [69]. Hence, sarcopenia should not be considered only a mere prognostic marker of therapeutic success, but an independent factor that constantly affects the prognosis and quality of life of these patients.

Whereas nowadays, CT scans and MRI represent the most reliable imaging techniques to assess the presence of this condition, the high costs, the exposure to ionizing radiation and the low availability make these techniques unsuitable for routinary use in follow-up. MUS could therefore represent a useful alternative to perform easy, cheap and repeatable evaluations.

The literature gives plenty of evidence on the role of CT parameters in this context, while the role of MUS in liver disease has been mainly studied in cirrhosis [35,53]. However, the paucity of studies on MUS for sarcopenia detection in HCC patients cannot currently support its use in daily practice as an alternative to the gold standard methods, such as CT. Moreover, the only data found in literature about this specific topic are controversial [36], due to the small cohort number and to potential bias linked to the degree of muscle wasting in the patients included. With the increase in MUS application in HCC populations, more data will be available to analyze its capacity as a potentially alternative method for sarcopenia assessment and support its reliability as a prognostic factor. Further insight upon this topic could be given from new technologies, like SWE or quantitative ultrasound. These technologies could integrate the use of MUS in HCC patients allowing for the better evaluation of muscle quality and derivation of numerical cut-offs to correlate with HCC stages, response to therapies and surgical risk.

## 6. Conclusions

MUS could be a future tool for assessing sarcopenia in HCC patients with the advantage of a cheap, easy-to-use and easily available technique. Although it has been recently studied in patients with chronic liver disease, with interesting results as a prognostic parameter, there is only little and controversial evidence on its applicability in patients with HCC. The evaluation of sarcopenia in this group of patients seems to be essential for outcome prediction, especially in patients undergoing surgery. Hence, further studies and analysis on larger cohorts are needed to explore the feasibility of MUS in this specific clinical context.

## Figures and Tables

**Table 1:** Comparison of CT, MUS, DXA and BIA advantages and disadvantages in sarcopenia diagnosis.

	Advantages	Disadvantages
**CT**	Standardized measure of SMI through the quantification of muscular area at the level of L3 vertebral body	Ionizing radiation High-cost equipment Trained specialists needed, including radiology technicians Limited possibility to follow up with patient without repeated exposure to radiation
**MUS**	Non-invasive No ionizing radiation Cost effective Can be performed bedside Can be performed repeatedly in patient’s follow-up Dynamic evaluation	Variability between operators Lack of a standardized measure for sarcopenia diagnosis
**DXa**	Whole-body scan Low-dose ionizing radiation Cost effective	Ionizing radiation Influenced by body thickness, hydration status and water retention
**BIA**	Low-intensity electrical current Cost effective	Influenced by hydration status Variability among health conditions, specific populations and different equipment

CT, computed tomography; MUS, muscle ultrasound; SMI, total skeletal muscle index; DXA, dual-energy X-ray absorptiometry; BIA, bioelectrical impedance analysis.

**Table 2:** Sarcopenia evaluation in HCC patients.

First Author, Year	Origin of the Study Population	No. of Patients	Imaging Technique	Parameters Evaluated
**Kobayashi et al., 2016 [56]**	patients undergoing hepatectomy for HCC	241	CT	IMAC and PMI
**Hamaguchi et al.,** **2016 [57]**	patients undergoing hepatectomy for HCC	492	CT	IMAC
**Yang J. et al.,** **2022 [10]**	patients undergoing hepatectomy for HCC	155	CT	SMI calculated by skeletal muscle cross-sectional area
**Yang S. et al.,** **2022 [59]**	patients undergoing hepatectomy plus adjuvant TACE for HCC	64	CT	SMI before and after hepatectomy plus adjuvant TACE
**Wu et al.,** **2023 [60]**	patients undergoing TARE for HCC	92	CT	TAM, PM and PS area
**Nam et al.,** **2023 [61]**	patients undergoing TARE for HCC	347	CT	SMI
**Loosen et al.,** **2023 [15]**	patients undergoing TACE for HCC	89	CT	SMI, MMA and BMD
**Chien et al.,** **2022 [62]**	patients undergoing TACE for HCC	260	CT	PM
**Kobayashi et al.,** **2018 [63]**	patients undergoing transarterial treatments for HCC	102	CT	SMI
**Takada et al.,** **2018 [65]**	patients undergoing therapy with sorafenib for HCC	214	CT	SMI
**Badran et al.,** **2020 [66]**	patients undergoing therapy with sorafenib for HCC	262	CT	SMI
**Antonelli et al.,** **2018 [67]**	patients undergoing therapy with sorafenib for HCC	96	CT	SMI
**Hiraoka et al.,** **2017 [68]**	patients undergoing therapy with sorafenib for HCC	93	CT	PMI
**Imai et al.,** **2019 [64]**	patients undergoing therapy with sorafenib for HCC	61	CT	SMI
**Cheng et al.,** **2020 [69]**	patients experimenting therapeutic failure with sorafenib for HCC	385	CT	TPMT/BH
**Imai et al.,** **2023 [71]**	patients undergoing therapy with lenvatinib or sorafenib for HCC	77	CT	SMI and SATI
**Oura et al.,** **2023 [24]**	patients undergoing therapy with Atezolizumab/Bevacizumab for HCC	64	GS BIA	GS SMI
**Hiraoka et al.,** **2023 [74]**	patients undergoing therapy with Atezolizumab/Bevacizum ab for HCC	229	CT	SMI
**Uojima et al.,** **2020 [72]**	patients undergoing therapy with lenvatinib for HCC	100	CT	SMI
**Luo et al.,** **2023 [73]**	patients undergoing combination therapy with immune checkpoint inhibitors and tyrosine kinase inhibitors	124	CT or MRI	PMI
**Xiong et al.,** **2023** **[70]**	patients undergoing immune checkpoint inhibitors for HCC	74	CT	SMI and SATI
**Yang J.-F. et al.,** **2022** **[75]**	patients undergoing stereotactic body radiotherapy for HCC	137	CT	SMI
**Sakai et al.,** **2022** **[36]**	patients with HCC	30	US CT	MT of the gastrocnemius and soleus and SMI

HCC, hepatocellular carcinoma; US, ultrasound; IMAC, intramuscular adipose tissue content; PMI, psoas muscle mass index; SMI, skeletal muscle index; MT, muscle thickness; TAM, total abdominal muscle; PM, psoas muscle; PS, paraspinal muscle; SATI, subcutaneous adipose tissue index; TARE, trans-arterial radioembolization; MMA, median muscular attenuation; BMD, bone mineral density; GS, grip strength; BIA, bioelectrical impedance analysis; TPMT, transverse psoas muscle thickness; BH, body height.

## Data Availability

These data were derived from the following resources available in the public domain: Medline via PubMed.
